# Mass fluctuation in breeding females, males, and helpers of the Florida scrub-jay *Aphelocoma coerulescens*

**DOI:** 10.7717/peerj.5607

**Published:** 2018-09-13

**Authors:** Marco Cucco, Reed Bowman

**Affiliations:** 1 DISIT, University of Piemonte Orientale, Alessandria, Italy; 2 Avian Ecology Program, Archbold Biological Station, Venus, FL, USA

**Keywords:** Florida scrub-jay, Mass variation, Electronic balance, Helpers, Cooperative breeder, Reproduction, Parents and helpers, Daily

## Abstract

Much evidence suggests that birds actively regulate their body mass reserves relative to their energy needs. Energy requirements during reproduction may differ in relation to sex-specific behavioural roles or, in the case of cooperative breeders, breeders relative to helpers. We measured body mass of free-living Florida scrub-jays throughout the nesting season by training them to land on an electronic balance. Jays exhibited a pattern of diurnal linear mass gain, from morning to afternoon. Day-to-day mass fluctuations, defined as the difference between mass on two consecutive days, were small (>80% were within 2 g, less than 3% of the mass of an adult bird) for all classes of jays: female breeders, male breeders and prebreeding helpers. The jays, which live in subtropical south-central Florida, did not exhibit changes in day-to-day mass fluctuation relative to weather or climate variables or calendar date. Day-to-day mass fluctuations influenced mass fluctuation between the following third and fourth days. These changes were usually compensatory, indicating that jays are able to regulate their body mass on a short-term basis, despite strong differences in their roles in reproduction. During reproduction, jays have a relatively predictable and abundant food supply, thus the appropriate strategy may be to maintain a stable body mass that balances some energy reserves against maintaining a low body mass for efficient flight, as required during reproduction.

## Introduction

Changes in body mass of birds should reflect a trade-off between costs and benefits of energy storage (reviewed in Witter & Cuthill, 1993). Costs of energy storage include a reduction in flight efficiency and thereby increased risk of predation (Lima, 1986; Rogers, 1987; Gosler, Greenwood & Perrins, 1995); benefits include lower probabilities of starvation and thermal stress, as well as a higher likelihood to survive in case of diseases and their related periods of pathogen-induced anorexia (Speakman, 2018). Much evidence suggests that birds actively regulate energy reserves relative to their needs (reviewed in Witter & Cuthill, 1993; Witter, Swaddle & Cuthill, 1995; Thomas, 2000), increasing reserves as food resources become more unpredictable (e.g., Bednekoff & Krebs, 1995; Ekman & Hake, 1990; Hurly, 1992; Ekman & Lilliendahl, 1993; but see Acquarone et al., 2002; Cucco et al., 2002; Boon, Visser & Daan, 1999) or predictably scarce, as when overnight temperatures decrease (e.g. Ekman & Hake, 1990). The ability of birds to regulate their body mass might be compromised during periods of elevated energy expenditure, as during the feeding of dependent young. The reproductive stress hypothesis invokes this idea to explain the mass loss during breeding (Bryant, 1979; Moreno, 1989). Alternately, it has been suggested that birds might deliberately maintain a lower mass to save energy during frequent flight as when flying back and forth while feeding young (flight adaptation hypothesis; Freed, 1981; Norberg, 1981).

In several studies body mass was positively correlated with fat reserves (see Witter & Cuthill, 1993), therefore fluctuations in body mass may provide insight into the trade-off between costs and benefits of fat storage on a daily basis. Most mass-regulation studies have looked at mass change within one day or change in body mass from before and after some experimental manipulation. We propose that the amplitude and pattern of day-to-day fluctuations in body mass over several days also can provide insight into management of energy reserves by birds.

Most mass-regulation studies have focused on temperate-zone birds that experience food shortages in winter (Lehikoinen, 1987; except Acquarone et al., 2002; Cucco et al., 2002, which looked at temperate zone birds in spring). Little is known about variation in body mass of birds living in tropical areas. Limited evidence suggests that under relatively uniform tropical conditions, seasonal variation in body mass of residents is small (Ward, 1969; Clark Jr, 1979; Nwaogu et al., 2017). In subtropical Florida scrub-jays, body mass varies little throughout the year (Woolfenden, 1978). It may be important for birds living in a mild climate and experiencing relatively predictable foraging conditions to maintain a stable and low body mass that is optimal for flight efficiency, because conditions in which energy reserves are useful are infrequent.

We measured the body mass of free-living Florida scrub-jays during the breeding season in south central Florida, USA. Florida scrub-jays breed cooperatively and maintain permanent, all-purpose territories (Woolfenden & Fitzpatrick, 1996). A strict dominance hierarchy exists within scrub-jay families in which males dominate females and, within each sex, breeders dominate helpers (Woolfenden & Fitzpatrick, 1977). A recent study on long term mass variation for females and males breeders throughout the breeding season showed that both sexes lost mass during the period of nestling care, but not when feeding fledglings. Although post-fledging is a period of peak effort (Brand & Bowman, 2012) this is also the period during which contributions by helpers is at its highest, suggesting that their contributions might buffer mass losses by breeders. Despite this, few studies have examined mass loss in non-breeding helpers of cooperative species. We examined variation in day-to-day mass fluctuation relative to social class (female breeder, male breeder, or prebreeding helper), stage of the breeding cycle (building, incubation, dependent nestlings, dependent fledglings) and fortnight of the breeding season. We sought to examine whether mass fluctuations were greater in birds lower in the social dominance hierarchy (Krams, 2002), and in the periods of higher energy demand, i.e., when nestling feeding occurrs (Moreno, 1989). We tested for the ability of individuals to maintain a stable mass by looking at the effect of body mass fluctuation between two consecutive days on fluctuation between the following third and fourth day. Beside the energy demands of parental effort during reproduction, bird mass could be influenced by local factors, such as predator presence or food abundance (Krams, 2000; Krams, 2002), and by daily weather conditions (Wiley & Ridley, 2016). We do not quantify variation in predator abundance among different scrub-jay territories. Most of the study area is well managed using prescribed fire. We do know that predator abundance increases and jay survival and reproduction declines with time since fire, but virtually all of our birds occupy habitats that are earlier than this successional decline in demography. From this we assume that predation risk is relatively evenly spread among all the territories. To test for possible short-term effects of climatic stress on mass (Lima, 1986), we also investigated whether day-to-day fluctuation in mass were related to any of six weather variables collected in the study area.

## Materials & Methods

This study was conducted at Archbold Biological Station in south-central Florida (27°10′N 81°21′W) as part of the long-term study of the Florida scrub-jay (Mumme et al., 2015; Fitzpatrick & Bowman; data collected under SFWS permit TE824723-9 issued to R. Bowman). Each jay in the population is uniquely marked with metal and plastic colour rings (Woolfenden, 1975), and all individuals are easily identifiable in the field. All birds are sexed genetically at only 11 days post-hatch, but later confirmed behaviourally; only females incubate and brood and give a characteristic “hiccup” vocalization (Woolfenden & Fitzpatrick, 1984). Prebreeding helpers, which remain in their natal territories for one or more years and help the breeders raise young, were distinguished from breeders by their histories and by behaviour, especially dominance-subordinance relationships (Woolfenden & Fitzpatrick, 1977). Data were collected during the breeding season of 1996, during which nesting began in mid-March, and almost all pairs had eggs by mid-April.

We collected mass measurements daily from 2 April to 9 June 1996. We placed electronic balances (accuracy 0.1 g) on the ground several tens of meters from the nest in numerous territories. The jays, which are remarkably tame to humans and accustomed to receiving peanut bits from us, were induced to perch on the balance by placing a few bits on the plate. The mass of peanuts provided daily to individual birds was less than 0.2 g, or about 0.3% of the mass of an adult jay (77 g; Woolfenden & Fitzpatrick, 1996). We used two methods for obtaining mass measurements. When a stable mass value was visible on the scale display, we read the scale directly. Otherwise, we inspected a series of mass values (approximately three per second) collected by a computer attached to the scale to find a stable mass value, defined as at least three consecutive identical values. All data were collected by the same observer (MC). We recorded only a single mass value for each visit of a bird to the scale. We obtained 438 mass values by reading the scale directly and another 494 from the attached computer for a total of 932 measurements (31 individuals: seven female breeders, 15 male breeders, and nine prebreeding helpers, sexes combined; mean = 30.1 ± 2.6 measurements per bird). For all visits, the observer recorded the identity of the bird and the time the mass value was obtained.

We divided date into five fortnight intervals (15 days) from 1 April through 15 June. We divided the breeding cycle into four stages: nest building and egg laying (hereafter referred to as building), incubation, nestlings, and dependent fledglings. Mass measurements obtained from birds after the failure of their last nest were excluded. We utilized mixed-effect models to compare mass values between the fortnight periods, or between the breeding stages. In the models, fortnight or breeding stage was inserted as fixed factor, and jay identity was inserted as a random effect to account for repeated measures (*lme4* package for R vers. 3.4.3, (R Development Core Team, 2017). Post-hoc comparisons with Tukey correction were computed by the package *multcomp*, and group means for fixed factors with confidence intervals were obtained by the package *lmerTest*.

We define day-to-day mass fluctuation as the difference in mass between two consecutive days. We define the amplitude of mass fluctuation as the absolute value of the day-to-day mass fluctuation. We examined day-to-day mass fluctuation relative to date and breeding stage separately for three social classes, e.g., female and male breeders and prebreeding helpers. Of the 932 mass values, 595 represented paired measurements of the same individuals on successive days. Because mass values taken over a sequence of days are potentially more correlated than masses that are further away in time (Ekstrom, 2012), we modelled the relationship between mass variation and elapsed days using mixed models with serial correlation in the *nlme* package (Pinheiro et al., 2018). In the models, the jay identity was inserted as a random term, while social class and breeding stage were inserted as fixed terms. We modelled both linear and quadratic effects.

We examined the influence of weather on day-to-day fluctuation in body mass using air temperature and rainfall: (1) maximum temperature on the day of weighing, (2) mean maximum temperature on the day of weighing plus the previous day, (3) minimum temperature on the day of weighing, (4) mean minimum temperature on the day of weighing plus the previous day, (5) amount of rainfall on the day of weighing, and (6) amount of rainfall on the day of weighing plus the previous day. Temperature and rainfall values were collected *in situ* and obtained from Archbold meteorological archives. Relationship between mass fluctuation and weather variables was tested both individually for each variable and in a multiple regression analysis, followed by stepwise backward and forward selection. Multivariate models were compared using Akaike information criterium’s values computed by the *nlme* package.

## Results

### Within-day increase in body mass

**Figure 1:**
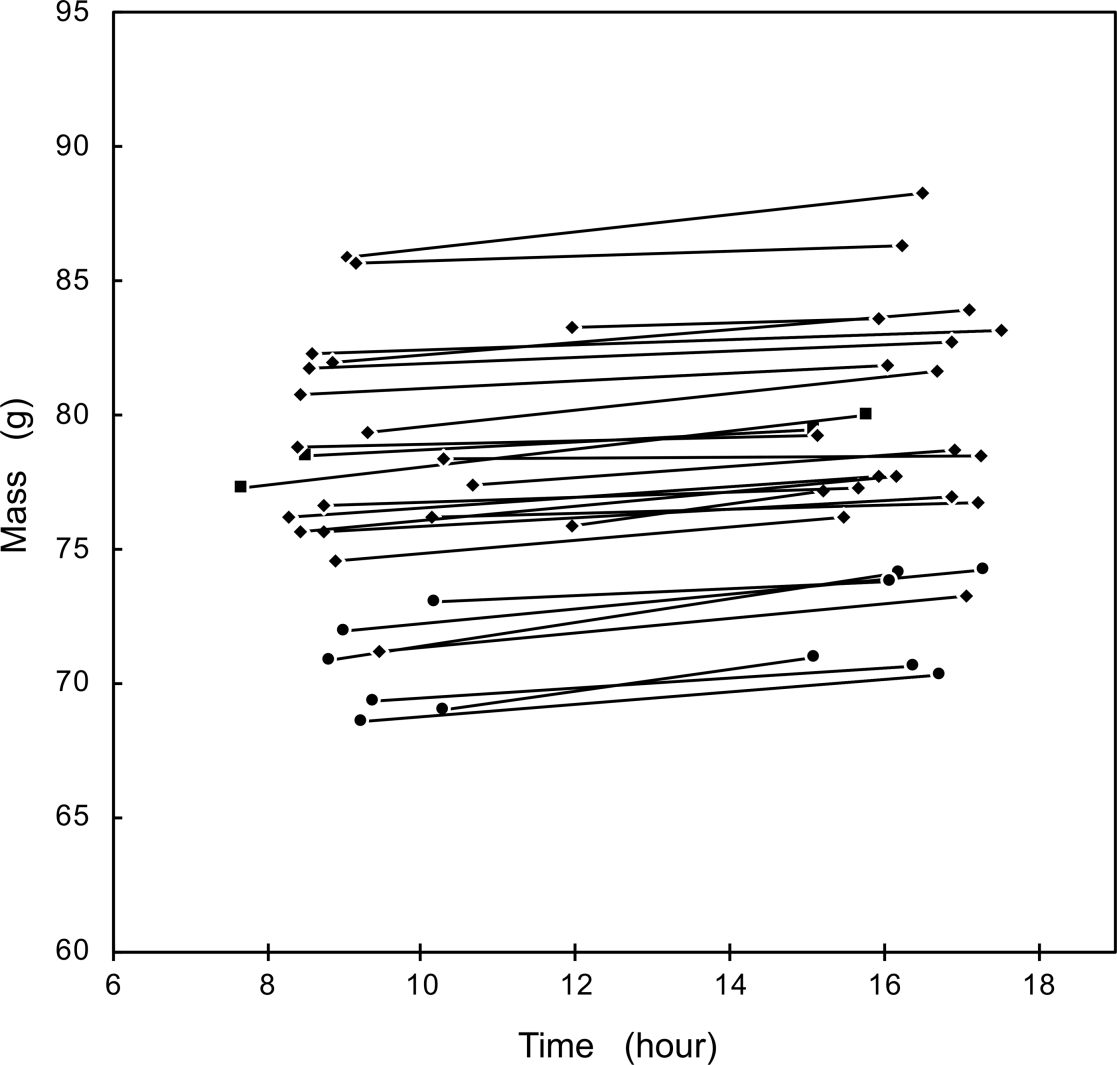
Mass change of Florida Scrub-Jays within a day. Circles represent female breeders; diamonds, male breeders; squares, helpers.

We observed an increase in body mass daily from morning to afternoon in 27 individuals that were weighed twice in the same day (Fig. 1). The average increase was 0.205 ± 0.117 g/h, with no significant differences between the three social classes (*F*_2,24_ = 2.035, *p* = 0.15). The mass increase during the examined central hours of the day was best explained by a linear increase (*t* = 1.691, *P* < 0.05), while the addition of a quadratic term was not significant (*t* = 0.354, *P* = 0.35).

### Mean body mass by fortnight and stage of the breeding cycle

The mean mass of jays was influenced by date and stage during the breeding season (Table 1). The mass of females was higher during the first fortnight and the first phase of the breeding stages, then mass values were significantly lower (mixed model ANOVA, five fortnights: *F*_4,177_ = 52.4, *p* < 0.001; three stages: *F*_2,162_ = 73.1, *p* < 0.001; Table 1). The mass of females was lower during the nestling stage (*t* = 5.74, *p* < 0.001, post hoc comparison with Tukey correction) and the fledgling stage (*t* = 7.69, *p* < 0.001) than during incubation. The mass of males and helpers showed significant differences in relation to date (five fortnights, males: *F*_4,511_ = 15.9, *p* < 0.003; helpers: *F*_4,244_ = 36.1, *p* < 0.001) and to breeding stage (three stages, males: *F*_2,439_ = 38.2, *p* < 0.001; helpers: *F*_2,206_ = 38.6, *p* < 0.001). However, mass in males showed no trend to decrease with time; their mass was highest during the middle fortnight (*t* = 3.38, *p* < 0.006) and middle stage (nestlings: *t* = 2.89, *p* < 0.004). The mass of helpers was highest in the third and fifth fortnights (*t* = 5.02 and *t* = 3.19, *p* < 0.01) and in the last two stages (nestlings, *t* = 5.77, *p* < 0.001; fledglings, *t* = 5.15, *p* < 0.001).

**Figure 2:**
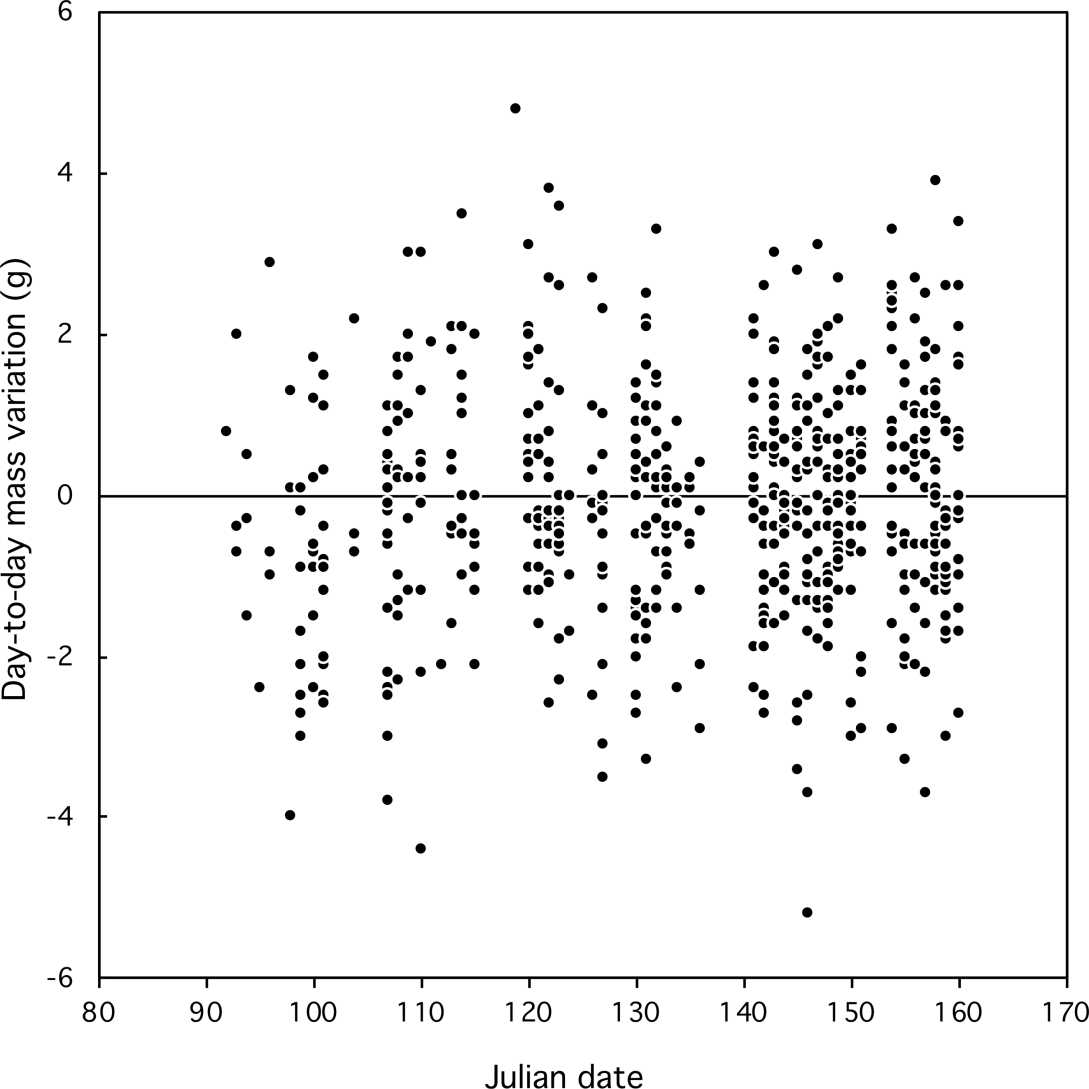
Day-to-day fluctuations in body mass of Florida Scrub-Jays relative to date.

**Table 1:** Body mass (g, mean ± s.e.) of Florida scrub-jays relative to fortnight and breeding stage. Differences within a class among time periods are indicated by different superscript letters.

Period	Females	Males	Helpers
Fortnight			
April 1–15	75.4 ± 1.59^a^	78.0 ± 1.17^a^	73.9 ± 0.98^a^
April 16–30	74.0 ± 1.57^b^	77.5 ± 1.16^a^	74.9 ± 0.96^a^
May 1–15	73.7 ± 1.56^b^	78.0 ± 1.16^b^	75.9 ± 0.95^b^
May 16–31	72.0 ± 1.56^c^	77.2 ± 1.16^a^	74.9 ± 0.93^a^
June 1–15	71.9 ± 1.57^c^	77.7 ± 1.16^a^	76.1 ± 0.97^b^
Breeding stage			
Incubation	76.6 ± 1.16^a^	78.3 ± 1.15^a^	73.7 ± 1.02^a^
Nestlings	74.2 ± 1.08^b^	77.0 ± 1.14^b^	75.6 ± 1.01^b^
Fledglings	72.1 ± 1.06^c^	77.5 ± 1.15^c^	75.8 ± 1.01^b^

**Figure 3:**
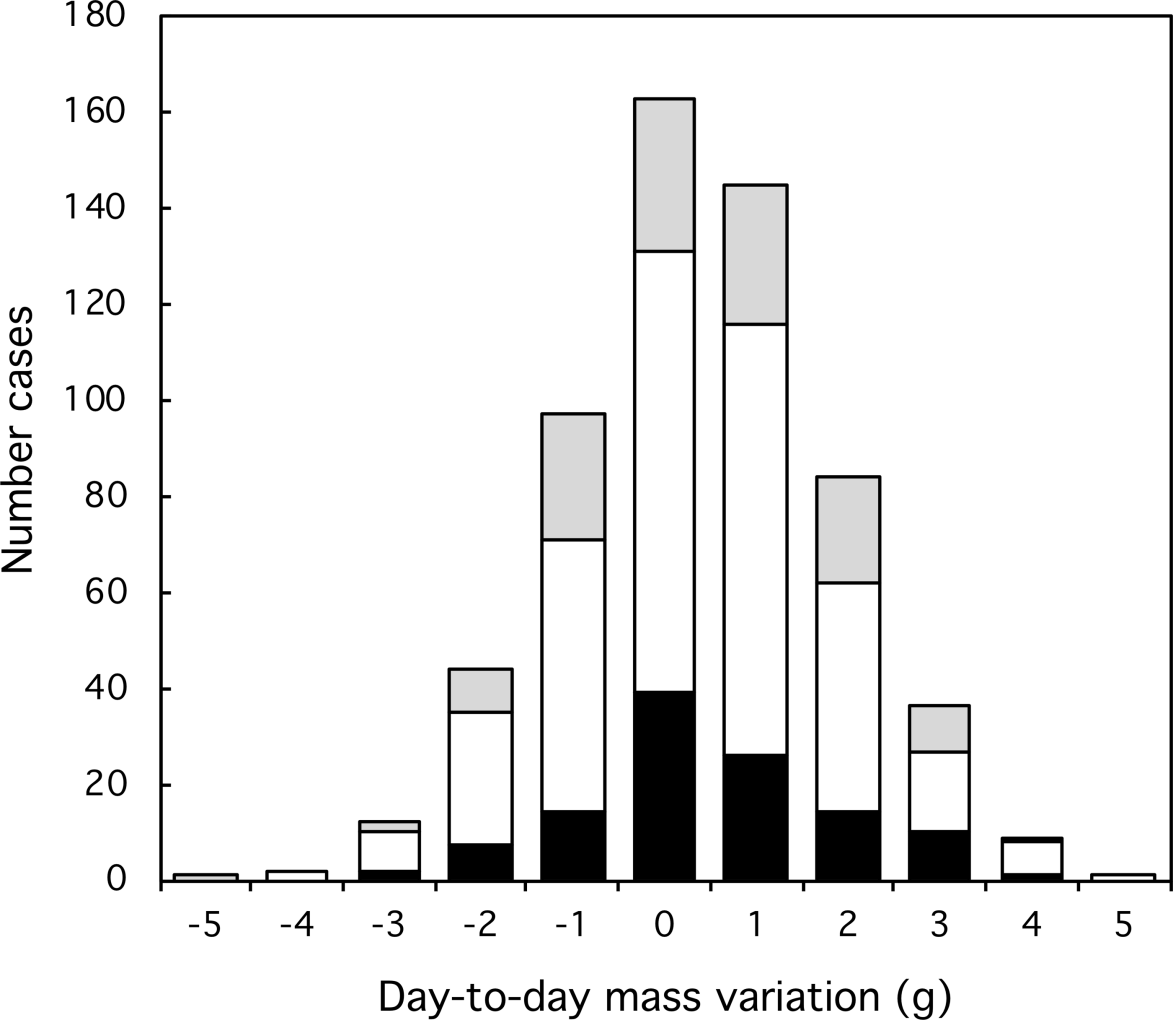
Variation and extent of day-to-day mass fluctuations in three classes of Florida Scrub-Jay. Black: females; white: males; grey: helpers.

### Day-to-day fluctuations in mass

Most day-to-day fluctuations in mass were small, most (80%) within 2 g, which is less than 3% of the mass of an adult bird (77 g). The median fluctuation was 0.2 g with a range from −4.0 to +6.4 g. The amplitude of mass fluctuations (Fig. 2) did not vary by date (Mixed-effect model with serial correlation; Julian date: *t* = 0.703, *p* = 0.48; quadratic term Julian^2^: *t* =  − 0.736, *p* = 0.46; scrubjay identity inserted as a random effect). We found no difference in the mean day-to-day mass fluctuation (Fig. 3) or the amplitude of mass fluctuations between females, males, and helpers (*F*_2,355_ = 0.28, *p* = 0.76; *F*_2,355_ = 1.27, *p* = 0.28; respectively).

Day-to-day mass fluctuation did not vary by fortnight for females (five fortnights: *F*_4,62_ = 0.57, *p* = 0.68) and helpers (*F*_4,80_ = 0.70, *p* = 0.59; Supplemental Information 1). Day-to-day mass fluctuations varied for males (*F*_4,201_ = 2.46, *p* = 0.05), but only the first (April 1–15; $\bar {x}=-0.58\pm 0.42$ SD) and last (June 1–15; $\bar {x}=0.60\pm 0.19$ SD) fortnights were significantly different (Tukey’s multiple comparisons, *p* = 0.03). The amplitude of mass fluctuations did not vary by fortnight for females (*F*_4,62_ = 0.73, *p* = 0.58), males (*F*_4,201_ = 1.09, *p* = 0.36) or helpers (*F*_4,80_ = 1.17, *p* = 0.33; Supplemental Information 2). Day-to-day mass fluctuation did not differ between breeding stages (four stages: *χ*^2^ = 1.39, *p* = 0.22), for females (*χ*^2^ = 6.41, *p* = 0.09), males (*χ*^2^ = 3.56, *p* = 0.31) or helpers (*F*_3,77_ = 2.31, *p* = 0.08; Supplemental Information 1). The amplitude of mass fluctuations did not vary by breeding stage for helpers (*F*_3,77_ = 0.13, *p* = 0.95). However, the amplitude of mass fluctuations was significantly higher for both females (*χ*^2^ = 8.32, *p* = 0.04) and males (*F*_3,176_ = 4.75, *p* = 0.003) during the building stage than for any other breeding stage (Tukey’s test, *p* < 0.05 for all comparisons, Supplemental Information 2).

Day-to-day fluctuation in mass of a scrub-jay was positively associated with the maximum temperature on the first day of weighing, as well as with maximum temperature of the first and second days of weighing (Table 2). Day-to-day fluctuation in mass was not associated with minimum temperatures, nor with rainfall. A multivariate analysis of all climatic variables and period of the breeding cycle or class (female breeder, male breeder, or helper) resulted in a final model including only the two maximum temperature values. This result was consistent with findings from the single-factor correlations.

**Table 2:** Correlations between weather variables and day-to-day mass fluctuations in Florida scrub-jays.

Variables	Estimate	Std. Error	*t* value	*P*
Single weather variables				
1 day maximum temperature	0.0339	0.0109	3.109	0.002**
2 days maximum temperature	0.0245	0.0124	1.972	0.049*
1 day minimum temperature	0.0076	0.0077	0.993	0.32
2 days minimum temperature	0.0048	0.0094	0.512	0.61
1 day rain	0.0128	0.1222	0.105	0.92
2 days rain	0.0282	0.0899	0.314	0.75
Stepwise complete model, AIC = 475.7				
Intercept	−2.0743	1.2185	−1.702	0.09
Julian date	−0.0012	0.0049	−0.247	0.80
Breeding stage	−0.0015	0.0026	−0.566	0.57
Status [helper]	−0.0522	0.1980	−0.264	0.79
Status [male]	-0.1315	0.1724	−0.762	0.45
^a^1 day maximum temperature	0.0683	0.0249	2.747	0.006**
^a^2 days maximum temperature	−0.0342	0.0278	−1.228	0.22
1 day minimum temperature	0.0087	0.0213	0.411	0.68
2 days minimum temperature	−0.0197	0.0263	−0.748	0.45
1 day rain	−0.0038	0.1728	−0.022	0.98
2 days rain	0.0829	0.1339	0.619	0.54

**Notes.**

a Stepwise selected variables (forward and backward selection) AIC = 461.9.

### Regulation of body mass

**Figure 4:**
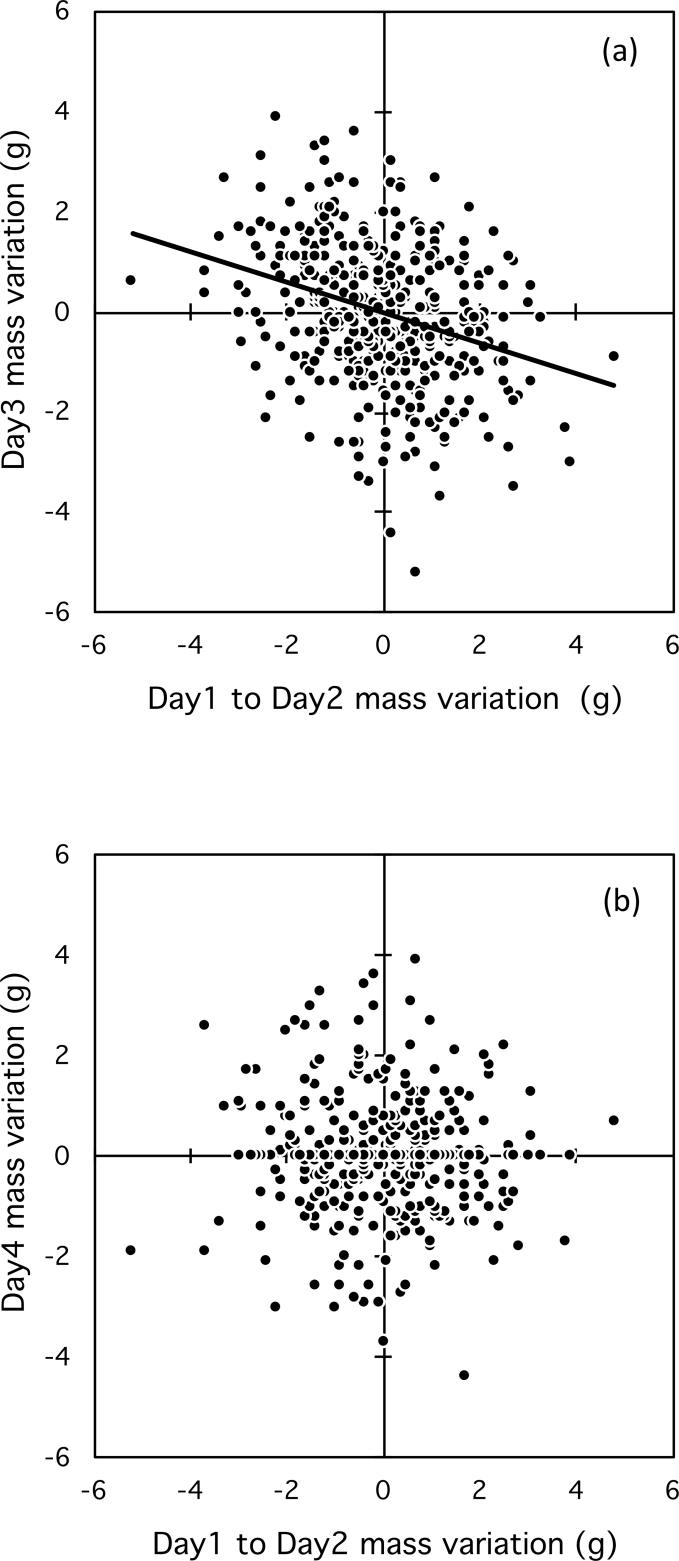
Relationship between mass fluctuation between two consecutive days and the second and third day (A) and the third and fourth day (B).

Day-to-day mass fluctuation influenced mass fluctuation between the second and third consecutive days (*F*_1,236_ = 22.2, *p* < 0.001, ${r}_{\mathrm{adj.}}^{2}=0.082$). Usually the mass fluctuation from the second to third day was compensatory (Fig. 4A); however, the influence had only an immediate, short-term effect, and disappeared by the fourth consecutive day (Fig. 4B; *F*_1,129_ = 0.2, *p* = 0.65, ${r}_{\mathrm{adj.}}^{2}=-0.006$). The effect of day-to-day mass fluctuation on mass fluctuation between the second and third days was significant for all classes: female breeders (*F*_1,44_ = 7.81, *p* = 0.008, ${r}_{\mathrm{adj.}}^{2}=0.131$), male breeders (*F*_1,142_ = 7.65, *p* = 0.006, ${r}_{\mathrm{adj.}}^{2}=0.044$), and helpers (*F*_1,45_ = 5.46, *p* = 0.02, ${r}_{\mathrm{adj.}}^{2}=0.088$). Mixed-effect models with serial correlation showed that mass fluctuations over three-day series (*t* =  − 0.658, *p* = 0.51) did not differ from those over a four-day series (*t* =  − 0.835, *p* = 0.40); Supplemental Information 3).

## Discussion

### Temporal patterns of mass variation

Florida scrub-jays exhibited a pattern of diurnal mass gain frequently observed in other diurnal birds (reviewed in Clark Jr, 1979; Haftorn, 1989; Ekman & Lilliendahl, 1993; but see Manu & Cresswell, 2013). Birds weighed the least in the morning, presumable because of nighttime fasting, and gained weight throughout the day. Birds in all social classes—female and male breeders and helpers—exhibited this pattern, although only one helper was measured multiple times within a single day. The 0.205 g gain in mass per hr is great enough to warrant caution whenever mass measurements taken at different times of day are compared. In this study we accordingly inserted the time of the day in all models comparing mass measurements. Time-adjusted measurements of the same birds taken on consecutive days, possible in this study because individuals were trained to land on an electronic balance and therefore were measured without capture are a useful alternative to repeated measurements of birds at the same time of day, for example dawn or dusk, employed in many laboratory studies (e.g., Thomas, 2000), but very difficult to obtain and very stressful for free-living birds (Carpenter, Paton & Hixon, 1983; MacLeod et al., 2005; MacLeod & Gosler, 2006; Moiron, Mathot & Dingemanse, 2018).

Breeding stage and calendar date (fortnights) affected the mass of female breeders. Females exhibited the common pattern in passerines of mass loss between the incubation and nestling stages (reviewed in Moreno, 1989). Mass during the fledgling stage also was lower than mass during the incubation stage. Male breeders did not show a tendency to decrease in mass during the breeding period. Their weigh fluctuated and showed some significant difference when comparing different stages or fortnights, but the differences were small (<1 g). The patterns of mass variation for female and male breeders in this study are consistent with mass data for an adjacent study population of the Florida scrub-jay collected over three years during the building, incubation and nestling stages (Schoech, 1998) and to data collected in the same population in different years (Brand & Bowman, 2012). However, in these studies the patterns of mass variation in helpers were never examined in detail before. Helpers exhibited an increase of mass in the last part of the breeding period, which is somewhat surprising given their energetically demanding contributions to feeding of nestlings (Stallcup & Woolfenden, 1978) and fledglings (McGowan & Woolfenden, 1990). This suggests helpers may be employing a bet hedging strategy by providing help to their parents thereby increasing their indirect fitness, yet at the same time attempting to maintain or even gain mass thereby not potentially reducing their longer-term survival prospects. Several studies documented costs associated with helping in cooperative breeders (Reyer, 1984; Heinsohn & Cockburn, 1994; MacLeod & Clutton-Brock, 2015). Florida scrub-jay helpers might be attempting to minimizing these costs to protect their long-term direct fitness potential by surviving to become breeders.

### Day-to-day fluctuations in mass

Day-to-day fluctuations in mass might be stochastic variation or might reflect difficulty in maintaining body mass, such as during unfavorable foraging conditions, cold weather, or a period of elevated energy expenditure. Consistent with the relative stability of Florida scrub-jays’ average mass during this study, most day-to-day fluctuations in mass were small, and overall did not vary between male breeders, female breeders and helpers despite their different breeding-season activities. Mass fluctuations varied significantly relative to season only for male breeders, and only between two fortnights, but these differences were small (−0.58 g during the first fortnight compared to 0.60 g during the last fortnight) and were not reflected by differences in average mass of males between fortnights. Also, the amplitude of mass fluctuations did not vary by fortnight.

In Florida scrub-jays, female breeders perform all incubating and brooding, but feed nestlings at a relatively low rate compared to the male breeder. Male breeders feed the female during incubation and brooding, and feed nestlings at a high rate. Both males and females feed fledglings at an increasing rate that peaks about two weeks post-fledging (Woolfenden & Fitzpatrick, 1996; Stallcup & Woolfenden, 1978; McGowan & Woolfenden, 1990). We found that despite the different reproductive activities of Florida scrub-jays, day-to-day mass fluctuations did not differ among social classes in different stages of the breeding cycle. The amplitude of these fluctuations also did not differ between classes. However, the amplitude of mass fluctuations was higher for female and male breeders during the building stage than during any other stage. The building stage encompassed the prelaying period, so the large amplitude of mass fluctuations for females likely reflects mass gain during the building stage. The average amplitude and fluctuation are equal for this stage, indicating that all fluctuation measurements were positive. The larger amplitude for males during the building stage is more difficult to explain. The average fluctuation for this stage was smaller than the average amplitude, though it was still positive, indicating that day-to-day mass gains were partly offset by losses. No differences in amplitude existed for helpers.

Weather variables explained less than 2% of the total variation in day-to-day mass fluctuation. The lack of effect of daily minimum temperature on mass fluctuation suggests that during the study period the nocturnal energy expenditure of jays was not influenced by nighttime temperature. It is not surprising that Florida scrub-jays in the subtropical climate of south-central Florida, particularly in the spring months of April, May and June, would not exhibit fat storage patterns observed in birds that experience colder temperatures (e.g., Rogers, 1987; Rogers & Smith, 1993; review in Witter & Cuthill, 1993).

### Regulation of body mass

Day-to-day mass fluctuation explained 8% of the variation in mass on the third consecutive day. Female breeders, male breeders and helpers exhibited similar short-term mass regulation, with the effect strongest in female breeders (13% of variation explained). Small mass fluctuations over two days, combined with the ability to compensate for these fluctuations on the third day, indicate that during the breeding season scrub-jays are able to regulate their body mass on a short-term basis. A number of studies have found differences in mass regulation strategies between dominant and subordinate birds (Krams, 2000; Krams, 2002). Theory predicts that subordinates should carry greater fat reserves than dominants (Clark & Ekman, 1995; Gosler, 1996; Ratikainen & Wright, 2013) as insurance against poor foraging conditions, during which dominants would exercise their priority access to food. Some studies have found that the body mass of subordinates varies less than that of dominants, likely because smaller adjustments are needed to subordinates’ already larger reserves (in Willow Tits *Parus montanus*, Ekman & Lilliendahl, 1993; Great Tits *Parus major,* Gosler, 1996; and Greenfinches *Carduelis chloris*, Hake, 1996; but the opposite result in Coal Tits *Parus ater,* Polo & Bautista, 2002). In contrast, in Florida scrub-jays, although helpers are subordinate to male breeders, they exhibited similar day-to-day mass fluctuations, amplitude of fluctuations and short-term mass regulation. These previous studies were performed on dominance-structured winter flocks of genetically unrelated individuals in which dominants were able to monopolize food resources, leading to different strategies. In Florida scrub-jays, dominant and subordinate birds within groups are closely related. Jays tend to forage individually primarily on insects and, to a lesser extent, small reptiles, amphibians, and mammals all well hidden in the vegetation. This reduces the chance for dominant birds to monopolize food. In fall and winter, each jay feeds primarily on acorns, cached during the later summer. Each jay scatter-hoards 6-7000 individual acorns (DeGange et al., 1989). When caching, jays adopt behaviors that appear to minimize kelptoparsitism by dominant jays, suggesting that it may happen but is minimized within groups (Toomey, Bowman & Woolfenden, 2007). Thus year-round feeding patterns do not favor despotic control of food. Since breeder and helper jays both contribute to rearing young, it is not surprising that in this species, mass regulation strategies did not differ by social status.

Because the jays rarely experience severe climate or food shortages in spring (Woolfenden & Fitzpatrick, 1996), a strategy of fattening may be more of a disadvantage (Lima, 1986) than an advantage. Instead, for this species, the appropriate strategy seems to be maintain a stable body mass that effectively balance the need to maintain some energy reserves against the need for efficient flight and foraging, as required during reproduction.

## Conclusions

The mechanism for maintaining a stable body mass in Florida scrub-jays appears to operate on a daily basis, with birds experiencing a mass gain or loss over two consecutive days making a compensatory adjustment during the third day. Future studies could investigate whether or not scrub-jays are able to maintain such a short-term regulation of body mass in other seasons of the year, if this regulation can be supported in poor quality territories (Mumme et al., 2015; Fitzpatrick & Bowman, 2016) and in territories were food is less predictable (Schoech, Bowman & Reynolds, 2004).

##  Supplemental Information

10.7717/peerj.5607/supp-1 Supplemental Information 1 Supplemental material 1 Average day-to-day mass fluctuation (g, mean ±sd) ( *n* = number of independent data couplets measured during each fortnight or stage) of Florida scrub-jays relative to fortnight and breeding stage. Differences within each class among time periods are indicated by different superscript letter.

10.7717/peerj.5607/supp-2 Supplemental Information 2 Supplemental material 2 Average amplitude of day-to-day mass fluctuation (g, mean ± sd) (*n* = number of independent data couplets measured during each fortnight or stage) of Florida Scrub-Jays relative to fortnight and breeding stage. No significant differences existed between females, males, and helpers, for fortnight or for breeding stage; nor did differences exist within each sex among fortnights or stage.

10.7717/peerj.5607/supp-3 Supplemental Information 3 Supplemental material 3 Difference in mass along 3-days-long series, and 4-days-long series. Mixed models with serial correlation. The scrubjay identity was inserted as a random effect. Breeding stage and breeding class were inserted as fixed effects, and time elapsed between measures as a serial variable.

10.7717/peerj.5607/supp-4 Data S1 Raw data on mass increase within the same day Florida scrub jay mass variation within the same day.

10.7717/peerj.5607/supp-5 Data S2 Raw data on each individual daily mass Mass values for individual Florida scrub jays.

## Additional Information and Declarations

### Field Study Permissions

The following information was supplied relating to field study approvals (i.e., approving body and any reference numbers): Data were collected under USFWS permit TE824723-9 issued to R. Bowman.

### Data Availability

The following information was supplied regarding data availability: The raw data are provided in the Supplemental Files.
