# Supplementary material for: Mass fluctuation in breeding females, males, and helpers of the Florida scrub-jay Aphelocoma coerulescens
Source: PeerJ. 2018 Sep 13;6:e5607. doi: 10.7717/peerj.5607 (PMC6139246; doi:10.7717/peerj.5607)
Supplement: Supplemental Information 1 — Average day-to-day mass fluctuation (g, mean ±sd) ( n = number of independent data couplets measured during each fortnight or stage) of Florida scrub-jays relative to fortnight and breeding stage. Differences within each class among time periods are indicated by different superscript letter. [file peerj-06-5607-s001.docx]

Average day-to-day mass fluctuation (g, mean ± sd) (n = number of independent data couplets measured during each fortnight or stage) of Florida scrub-jays relative to fortnight and breeding stage. Differences within each class among time periods are indicated by different superscript letters.

---------------------------------------------------------------------------------------------------------------------

Period Females Males Helpers

---------------------------------------------------------------------------------------------------------------------

Fortnight

April 1-15 -0.68 ± 1.68 (4) -0.58 ± 1.85 (19)^a^ -0.43 ± 1.24 (10)

April 16-30 0.06 ± 1.35 (13) 0.39 ± 1.65 (37)^a, b^ 0.44 ± 1.85 (13)

May 1-15 -0.16 ± 1.61(16) 0.07 ± 1.50 (45)^a, b^ -0.02 ± 2.07 (17)

May 16-31 0.10 ± 1.11 (20) 0.14 ± 1.31 (60)^a, b^ 0.39 ± 1.32 (32)

June 1-15 0.40 ± 1.54 (14) 0.60 ± 1.25 (45)^b^ -0.16 ± 2.03 (13)

Breeding Stage

Building 2.45 ± 0.35 (2) 0.52 ± 2.17 (22) 0.84 ± 2.06 (13)

Incubating -0.80 ± 1.47 (4) 0.18 ± 1.36 (49) -0.06 ± 1.55 (21)

Nestlings -0.26 ± 1.55 (11) -0.24 ± 1.47 (38) 0.93 ± 1.38 (10)

Fledglings 0.08 ± 1.29 (50) 0.26 ± 1.39 (71) -0.19 ± 1.45 (37)

---------------------------------------------------------------------------------------------------------------------
