# Supplementary material for: Mass fluctuation in breeding females, males, and helpers of the Florida scrub-jay Aphelocoma coerulescens
Source: PeerJ. 2018 Sep 13;6:e5607. doi: 10.7717/peerj.5607 (PMC6139246; doi:10.7717/peerj.5607)
Supplement: Supplemental Information 2 — Average amplitude of day-to-day mass fluctuation (g, mean ± sd) (n = number of independent data couplets measured during each fortnight or stage) of Florida Scrub-Jays relative to fortnight and breeding stage. No significant differences existed between females, males, and helpers, for fortnight or for breeding stage; nor did differences exist within each sex among fortnights or stage. [file peerj-06-5607-s002.docx]

Average amplitude of day-to-day mass fluctuation (g, mean ± sd) (n = number of independent data couplets measured during each fortnight or stage) of Florida Scrub-Jays relative to fortnight and breeding stage. No significant differences existed between females, males, and helpers, for fortnight or for breeding stage; nor did differences exist within each sex among fortnights or stages.

---------------------------------------------------------------------------------------------------------------------

Period Females Males Helpers

---------------------------------------------------------------------------------------------------------------------

Fortnight

April 1-15 1.43 ± 0.85 (4) 1.53 ± 1.14 (19) 1.13 ± 0.58 (10)

April 16-30 0.98 ± 0.89 (13) 1.25 ± 1.13 (37) 1.55 ± 1.02 (13)

May 1-15 1.24 ± 0.99 (16) 1.16 ± 0.94 (45) 1.32 ± 1.55 (17)

May 16-31 0.86 ± 0.69 (20) 1.06 ± 0.78 (60) 1.10 ± 0.80 (32)

June 1-15 1.21 ± 0.98 (14) 1.07 ± 0.86 (45) 1.75 ± 0.92 (13)

Breeding Stage

Building 2.45 ± 0.35 (2) 1.87 ± 1.14 (22) 1.42 ± 1.68 (13)

Incubating 1.30 ± 0.88 (4) 1.01 ± 0.91 (49) 1.24 ± 0.89 (21)

Nestlings 1.33 ± 0.72 (11) 1.11 ± 0.99 (38) 1.19 ± 1.14 (10)

Fledglings 0.95 ± 0.87 (50) 1.13 ± 0.84 (71) 1.25 ± 0.74 (37)

---------------------------------------------------------------------------------------------------------------------
