# Supplementary material for: Mass fluctuation in breeding females, males, and helpers of the Florida scrub-jay Aphelocoma coerulescens
Source: PeerJ. 2018 Sep 13;6:e5607. doi: 10.7717/peerj.5607 (PMC6139246; doi:10.7717/peerj.5607)
Supplement: Supplemental Information 3 — Difference in mass along 3-days-long series, and 4-days-long series. Mixed models with serial correlation. The scrubjay identity was inserted as a random effect. Breeding stage and breeding class were inserted as fixed effects, and time elapsed between measures as a serial variable. [file peerj-06-5607-s003.docx]

**Difference in mass along 3-days-long series, and 4-days-long series.**

*Mixed models with serial correlation. The scrubjay identity was inserted as a random effect. Breeding stage and breeding class were inserted as fixed effects, and time elapsed between measures as a serial variable.*

---------------------------------------------------------------------------------------------------------------------

Variables Estimate Std. Error t value P

---------------------------------------------------------------------------------------------------------------------

**3 days long series**

AIC = 5378.5. Random effects (StdDev): intercept = 3.665; residual = 2.1105

Intercept 76.875 2.0951 36.693 0.001

Time (days) -0.068 0.1040 -0.658 0.51

Class[helpers] -2.080 2.5776 -0.807 0.43

Class[males] 2.407 2.3621 1.019 0.32

Stage[fledglings] -3.126 1.6038 -1.949 0.052

Stage[incubation] 1.619 1.7944 0.902 0.37

Stage[nestlings] -2.098 1.5187 -1.381 0.17

Class[helpers]:Stage[fledglings] 3.939 1.7715 2.224 0.026

Class[males]:Stage[fledglings] 2.457 1.6718 1.470 0.14

Class[helpers]:Stage[incubation] -2.757 1.8759 -1.470 0.14

Class[males]:Stage[incubation] -1.637 1.8340 -0.893 0.37

Class[helpers]:Stage[nestlings] 3.898 1.6710 2.333 0.02

Class[males]:Stage[nestlings] 0.223 1.5872 0.141 0.89

**4-days-long series**

AIC = 6668.1. Random effects (StdDev): intercept = 3.740; residual = 2.119

Intercept 76.102 2.07433 36.688 0.001

Time (days) -0.057 0.06881 -0.835 0.40

Class[helpers] -1.533 2.56617 -0.597 0.56

Class[males] 3.245 2.34755 1.382 0.18

Stage[fledglings] -2.139 1.51474 -1.412 0.16

Stage[incubation] 2.212 1.69198 1.307 0.19

Stage[nestlings] -1.201 1.44159 -0.833 0.41

Class[helpers]:Stage[fledglings] 3.038 1.66394 1.826 0.07

Class[males]:Stage[fledglings] 1.459 1.57321 0.928 0.35

Class[helpers]:Stage[incubation] -3.038 1.76452 -1.722 0.09

Class[males]:Stage[incubation] -2.251 1.72626 -1.304 0.19

Class[helpers]:Stage[nestlings] 3.129 1.57520 1.986 0.047

Class[males]:Stage[nestlings] -0.828 1.50050 -0.552 0.58

---------------------------------------------------------------------------------------------------------------------
